# Cost-effectiveness of active surveillance versus early surgery for thyroid micropapillary carcinoma based on diagnostic and treatment norms in China

**DOI:** 10.3389/fendo.2023.1166433

**Published:** 2023-08-16

**Authors:** Min Lai, Miao Miao Zhang, Qing Qing Qin, Yu An, Yan Ting Li, Wen Zhen Yuan

**Affiliations:** ^1^ The First School of Clinical Medicine, Lanzhou University, Lanzhou, China; ^2^ Department of Oncological Surgery, First Hospital of Lanzhou University, Lanzhou, China

**Keywords:** active surveillance (AS), early surgery, cost-effectiveness (CE), papillary thyroid microcarcinoma (PTMC), papillary thyroid cancer (PTC)

## Abstract

**Objectives:**

In this study, we compared the cost-effectiveness comparison of the active surveillance (AS) and early surgery (ES) approaches for papillary thyroid microcarcinoma (PTMC) from the perspective of the Chinese healthcare system.

**Methods:**

We performed a cost-effectiveness analysis using a Markov model of PTMC we developed to evaluate the incremental cost-effectiveness ratio of AS and ES. Our reference case was of a 40-year-old woman diagnosed with unifocal (<10 mm) PTMC. Relevant data were extracted after an extensive literature review, and the cost incurred in each state was determined using China Medicare data on payments for ES and AS. The willingness-to-pay threshold was set at ¥242,928/quality-adjusted life-year (QALY) gained. Sensitivity analyses were performed to account for any uncertainty in the model’s variables. Additional subgroup analyses were performed to determine whether AS was cost-effective when different initial monitoring ages were used.

**Results:**

ES exhibited an effectiveness of 5.2 QALYs, whereas AS showed an effectiveness of 25.8 QALYs. Furthermore, the incremental cost-effectiveness ratio for ES versus AS was ¥1,009/QALY. The findings of all sensitivity analyses were robust. Compared with ES, AS was found to be the cost-effective strategy at initial monitoring ages of 20 and 60 years, with an incremental cost-effectiveness ratio of ¥3,431/QALY and −¥1,316/QALY at 20 and 60 years, respectively. AS was a more cost-effective strategy in patients with PTMC aged more than 60.

**Conclusions:**

With respect to the norms of the Chinese healthcare system, AS was more cost-effective for PTMC over lifetime surveillance than ES. Furthermore, it was cost-effective even when the initial monitoring ages were different. In addition, if AS is incorporated into the management plan for PTMC in China at the earliest possible stage, a predicted savings of ¥10 × 10^8^/year could be enabled for every 50,000 cases of PTMC, which indicates a good economic return for future management programs. The identification of such nuances can help physicians and patients determine the best and most individualized long-term management strategy for low-risk PTMC.

## 1 Background

Papillary thyroid cancer accounts for 70% to 90% of thyroid malignancies and is characterized by slow growth and low invasiveness. Globally, the incidence of thyroid cancer has increased significantly in the past three decades (1) and continues to increase in the younger population (2). Meanwhile, the mortality of thyroid cancer has remained relatively stable at low levels or has decreased, almost worldwide (3). Among the various cancer types, papillary thyroid microcarcinoma (PTMC) (PTC < 1 cm) incidence showed the most rapid growth rate and the largest proportion (4). The drastic increase observed in PTMC incidence is generally attributed to the advanced and widespread use of diagnostic technologies such as high-resolution ultrasonography and fine-needle aspiration cytology (FNAC) (5, 6). Some investigators have suggested a diagnostic epidemic rather than an disease epidemic for PTMC—an accurate diagnosis of “cancer” that does not eventually manifest as symptoms or lead to death (7). PTMC overdiagnosis is also an example of medical service overuse, which increases health service and governmental expenditure (8). China is rapidly transitioning to a higher socioeconomic status. If the current growth rate continues, the overtreatment and burden of thyroid cancer may increase further. Additionally, overtreatment is an almost inevitable product of overdiagnosis, an aspect that has attracted great attention from medical establishments and the society at large. Therefore, identifying a cost-effective and sustainable strategy is a major priority for PTMC management in China.

Initial management measures involve weighing the risks and benefits of proposed treatment strategies. Surgery was a major priority for the management of low-risk PTMC before 2010 in various countries. In 2016, the Chinese Thyroid Oncology Society reached a consensus on the issue (9), stating that immediate surgical resection is the preferred treatment for patients with PTMC. Lin et al. (10) showed that surgery has a long-term economic advantage for young Australian patients with PTMC. However, even though the incidence of complications from thyroid cancer surgery is gradually decreasing, the complications are not completely avoidable (8). A multitude of unnecessary surgeries has led to an increase in the number of patients who require additional thyroxine supplementation and suffer from related complications. This may increase the physical and psychological burden of patients. Of note, the extent of thyroid cancer overdiagnosis and overtreatment is far from its peak (11, 12). The need for active early surgery (ES) treatment in low-risk PTMC has recently garnered attention (13).

Since 2010, the understanding of surgical treatment has changed in different countries. Active surveillance (AS) is a surgical alternative for selected patients with PTMC, and there is an expanding disease spectrum in which AS could be implemented. Japan established the first edition of guidelines in 2010, which adopted AS as an option for low-risk PTMC (14). In 2020, White et al. (15) reported that no negative consequences were observed among patients who were awaiting surgery, and most patients did not exhibit significant disease progression after 10 years. AS is considered a safe and effective alternative to active ES in appropriately selected patients. The results, which have been replicated in other studies, suggest that most patients with PTMC can be treated safely with AS and do not require active ES (15–18). In the latest treatment guidelines, AS strategy has been incorporated as an acceptable alternative treatment strategy for low-risk PTMC (19–22). Although currently available Chinese guidelines recommend AS for PTMC in 2022 (22), a large body of prospective clinical work on the use of AS for PTMC and most conclusions were obtained from studies published in other countries. A cost-effectiveness analysis for low-risk PTMC in China has not been conducted to date, and evidence on PTMC management with AS is insufficient. Therefore, there remain doubts regarding whether AS should be used widely in China. Additional evidence is needed on the costs and effectiveness of AS strategy in developing countries like China.

To bridge this evidential gap, we assessed the cost-effectiveness of ES and AS strategies as management approaches for patients with PTMC. We believe our findings could facilitate decision-making for patients and surgeons.

## 2 Methods

### 2.1 Patient reference case scenario

To indicate the range of individuals most representative of the patient cohort with thyroid nodules detected, the reference case selected was of an otherwise healthy, 40-year-old woman with a biopsied unifocal PTMC without characteristics that would warrant hemithyroidectomy (such as an unfavorable location near the trachea or recurrent laryngeal nerve, or lymph node metastases) or risk factors that would require a more aggressive surgical approach by resection (such as a family history of thyroid cancer, neck irradiation, other tumors, or uncontrolled chronic disease). The patient, who was initially in the AS or ES state, underwent various changes and eventually entered a state corresponding to death (life expectancy: 80 years). The model cycle was of 1 year with 40 cycles. The reasons for selecting this as the base case were as follows: (1) This was similar to the base case used in previous cost-effectiveness analyses of thyroid cancer to simulate the cost-effectiveness of different treatment methods. (2) We aimed to focus on AS with respect to the duration of follow-up and assess whether AS is cost-effective based on the need for lifetime surveillance. In addition, the incidence of thyroid cancer is higher in women. Thus, we selected a 40-year-old female patient for the base case.

### 2.2 Model overview

**Figure 1:**
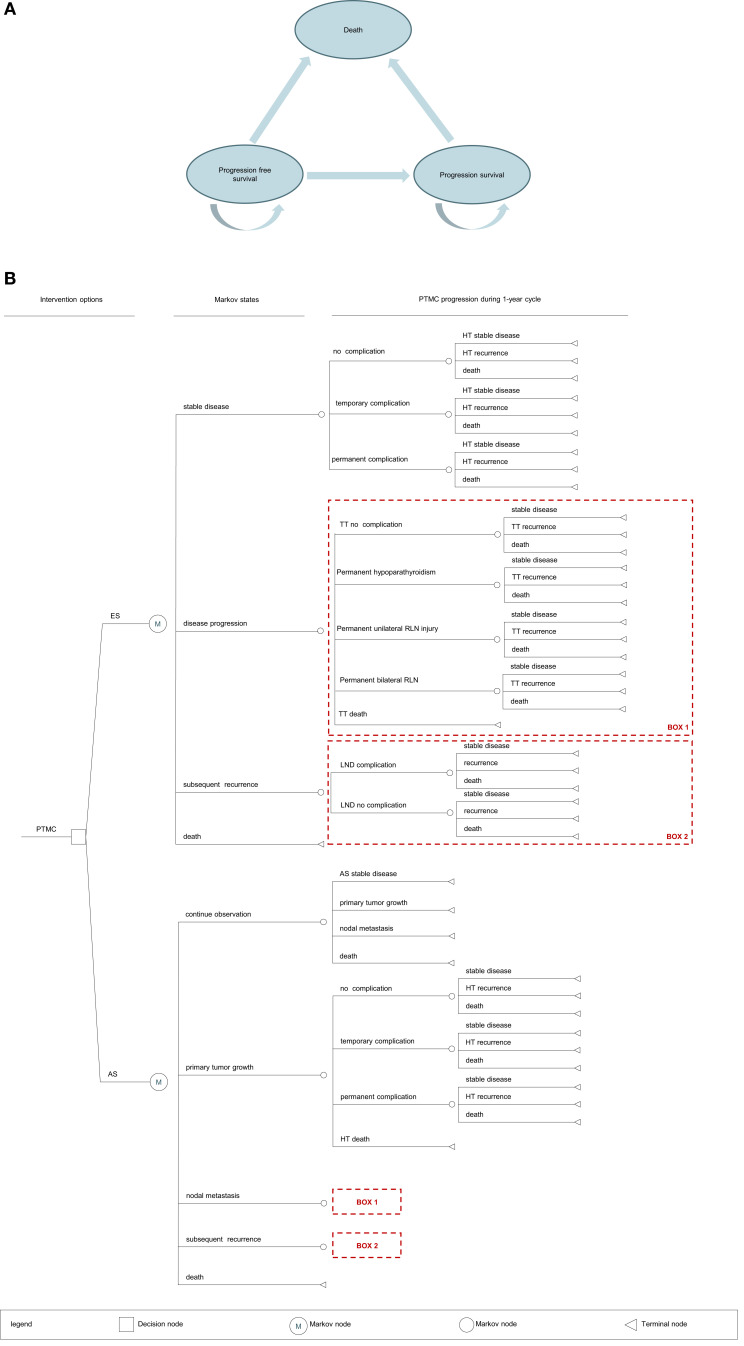
**(A)** Bubble plot. PTMC, papillary thyroid microcarcinoma; AS, active surveillance; ES, early surgery; HT, hemithyroidectomy; TT, total thyroidectomy; LND, lymph node dissection. **(B)** Markov model structure. PTMC, papillary thyroid microcarcinoma; AS, active surveillance; ES, early surgery; LND, lymph node dissection.

We provided evidence-based policy recommendations by developing a comprehensive and dynamic decision-analytic Markov model. The model was constructed using Tree Age Pro 2011 (**Figure 1**). A Markov decision tree provides a logical structure of decisions and potential events as they unfold over time. The Markov nodes for AS and ES represent a potential transition to new health states and include the following:

▪ Stable disease
▪ Disease progression (including primary tumor growth increase ≥3 mm, FNAC-confirmed lymph node metastasis in the new area)
▪ Lateral lymph node metastases
▪ Death

Quality-adjusted life-year (QALY) is a metric that reflects the length and health-related quality of life. It is calculated using the number of years lived and the utility score of a particular state of health. It is a dimensionless number between 1 (perfect health) and 0 (death).

### 2.3 Treatment characteristics and strategies

**Figure 2:**
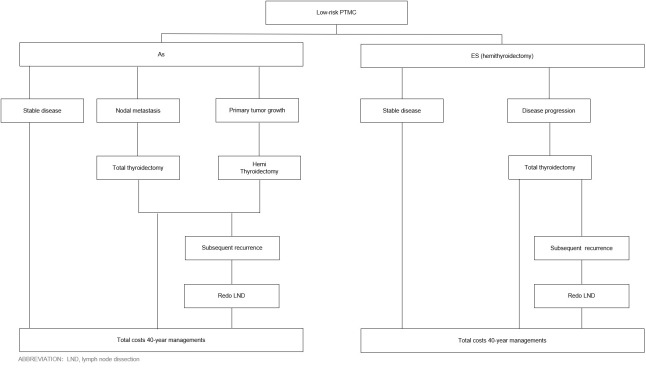
PTMC is based on ES and AS management processes. ICER, incremental cost-effectiveness ratio; AS, active surveillance; ES, early surgery; HT, hemithyroidectomy; TT, total thyroidectomy.

Patients undergoing AS were monitored when they still had cancer, whereas patients undergoing ES were monitored after cancer removal. If patients in the AS group developed a primary tumor growth, they underwent hemithyroidectomy + isthmectomy + unilateral central neck dissection. If patients in the ES group developed a new tumor in the remaining glandular lobes after surgery, they underwent total thyroidectomy + unilateral central neck dissection. If patients in either group developed central (group VI) lymph node metastases or lateral lymph node metastases, they underwent total thyroidectomy + central neck dissection or lateral lymph node dissection, respectively. In contrast, patients who did not develop metastases in the lymph node or other areas were monitored indefinitely (**Figure 2**).

Next, patients in both groups were subjected to a 40-year follow-up, which commenced when the patients were 40 and was conducted twice a year. Patients in both groups were monitored annually with a surveillance regimen comprising physician office visits, thyroid function and blood tests, neck/supraclavicular lymph node ultrasonography examinations, laryngoscopy, and other related tests. The tests were conducted every 6 months. Chest computed tomography scans and computed tomography neck enhancement scans were conducted once a year.

Permanent complications from hemithyroidectomy included permanent vocal cord palsy and hypothyroidism. Short-term complications included temporary vocal cord palsy. Complications from total thyroidectomy included permanent hypothyroidism, hypoparathyroidism, and unilateral/bilateral recurrent laryngeal nerve injury.

### 2.4 Sensitivity and scenario analyses

**Figure 3:**
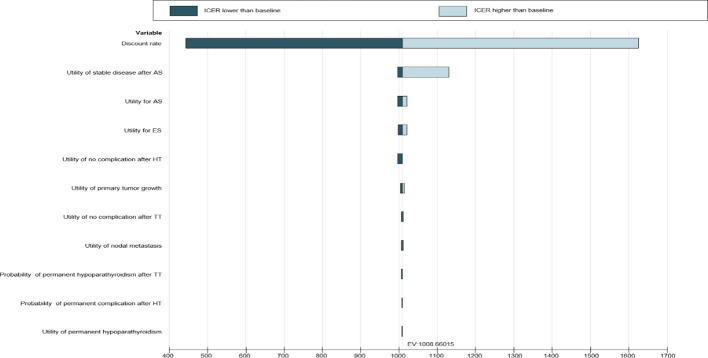
Tornado diagram–ICER–patients.

A series of sensitivity analyses were performed to explore how the results varied across a plausible range. The results of a deterministic sensitivity analysis are presented as tornado figures (**Figure 3**). One-way sensitivity analyses were performed to assess the impact of individual parameters in the model. In the univariable sensitivity analysis, transformation probability, health utility value, and cost change range were set to ±10%, and the discount rate range was set to 1% to 5% (23) (**Table 1**; **Supplementary Tables 2**, **3**).

**Table 1:** Inputs of the Markov model.

Input	Value	Distribution	Analysis range	Mean	Standard deviation	Country	Reference
Probability							
Permanent complication from HT	0.015	Beta	0.014-0.017	0.016	0.0008	Hong Kong, America	17, 24
Temporary complication from HT	0.015	Beta	0.014-0.017	0.016	0.0008	America	15
Recurrence after HT	0.004	Beta	0.0036-0.0044	0.004	0.0002	America	25
Recurrence after TT	0.008	Beta	0.007-0.009	0.008	0.0005	America	15
Stable disease after HT	0.684	Beta	0.616-0.752	0.684	0.035	America	26
Permanent unilateral RLN injury after TT	0.015	Beta	0.014-0.017	0.016	0.0008	America	15
Hypoparathyroidism after TT	0.089	Beta	0.080-0.098	0.09	0.005	America	15
Permanent bilateral RLN injury after TT	0.003	Beta	0.0027-0.0033	0.003	0.0002	America	15
Death after HT	0.002	Beta	0.0018-0.0022	0.002	0.0001	America	15
Death after TT	0.002	Beta	0.0018-0.0022	0.002	0.0001	America	15
Complication from redo LND	0.32	Beta	0.288-0.352	0.32	0.16	Hong Kong, America	17, 24
Probability nodule growth							
20-29	0.22	–	–			Japan	27
30-39	0.084	–	–			Japan	27
40-49	0.0380	–	–			Japan	27
50-59	0.0240	–	–			Japan	27
60-69	0.0003	–	–			Japan	27
70-79	0.0006	–	–			Japan	27
Probability nodal metastasis							
20-29	0.165	–	–			Japan	27
30-39	0.061	–	–			Japan	27
40-49	0.0380	–	–			Japan	27
50-59	0.0240	–	–			Japan	27
60-69	0.0068	–	–			Japan	27
70-79	0.0036	–	–			Japan	27
Health utilities							
AS stable disease	0.99	Beta	0.89-1	0.99	0.05	America	28
No complication after HT	0.99	Beta	0.89-1	0.99	0.05	America	25
Permanent complication after HT	0.63	Beta	0.57-0.69	0.63	0.032	America	29
Temporary complication after HT	0.627	Beta	0.56-0.69	0.627	0.032	America	15
Permanent bilateral RLN injury after TT	0.21	Beta	0.19-0.23	0.21	0.01	America	30
Utilities of Permanent hypoparathyroidism after TT	0.778	Beta	0.7-0.86	0.778	0.039	America	30
Permanent unilateral RLN injury after TT	0.6729	Beta	0.57-0.69	0.6279	0.032	America	30
Redo LND	0.56	Beta	0.5-0.62	0.56	0.029	America	29
LND complication	0.41	Beta	0.37-0.45	0.41	0.02	America	29
No complication after TT	0.83	Beta	0.75-0.91	0.83	0.042	America	15
Active surveillance	0.11	Beta	0.1-0.12	0.11	0.006	America	25
Disease progression	0.54	Beta	0.49-0.59	0.54	0.028	America	25
Early surgery	0.74	Beta	0.67-0.81	0.74	0.038	America	29
Nodal metastasis	0.25	Beta	0.23-0.28	0.25	0.013	America	15
Primary tumor growth	0.54	Beta	0.49-0.59	0.54	0.028	America	29
Recurrence	0.54	Beta	0.49-0.59	0.54	0.028	America	29
Discount rate	0.03		0-0.05			China	
Start age	20						

HT, hemithyroidectomy; TT, total thyroidectomy; RLN, recurrent laryngeal nerve; LND, lymph node dissection.

Probabilistic sensitivity analyses were conducted to explore uncertainties around model inputs by varying them simultaneously. Probabilistic sensitivity analyses were performed using Monte Carlo simulations with 1,000 iterations with different distributions, where the transition probabilities and utilities followed a beta distribution pattern and the cost followed a normal distribution pattern. The ranges and distribution patterns of the parameters used in the sensitivity analyses are shown in **Supplementary Table 3**. The results of the probabilistic sensitivity analyses are presented as scatter plots and cost-effectiveness acceptability curves (**Supplementary Figures 2**, **3**)

## 3 Model inputs

### 3.1 Probabilities

Estimates on the prevalence of complications from initial operations (total thyroidectomy and hemithyroidectomy) and reoperations (lymph node dissection) were derived from separate literature searches using specific terms like “recurrent laryngeal nerve,” “hypothyroidism,” “thyroidectomy,” “permanent complication,” and “temporary complication.” However, limited data are available on the transition probabilities from one stage to the next stage for Chinese patients with PTMC. Therefore, the probabilities of annual transition were inferred from published literature from other Asian countries, with a preference for data from countries with Chinese or associated populations (e.g., individuals from Hong Kong or Japan). If data for Asian individuals were unavailable, we used probabilities obtained for individuals from other regions. In studies where multi-year incidence was reported instead of the 1-year PTMC incidence, the 1-year incidence was calculated using the formula *r* = −log(1−*p*)/*t*, where *r* denotes the 1-year incidence and *p* represents the cumulative incidence over the length of the interval *t*. All transition probabilities from one health state to another took place in a 1-year cycle. For example, Miyauchi et al. (27) reported that the 10-year rates of primary tumor growth and regional lymph node metastases were 3.7% and 3.7%, respectively, for patients aged 40–59 years. These were converted to annual rates of 0.38% and 0.38%, respectively, for a Markov model with a 1-year cycle. Once the patients’ ages were changed, their probabilities for developing nodular growth and regional lymph node metastases were altered. Other transformation probability values were the same in all age groups (**Table 1**).

### 3.2 Utilities

The health utility value inputs used to calculate QALYs were obtained from the published literature. **Table 1** lists the utilities used in the model. Of note, the quality of life of patients undergoing AS for PTMC in a Chinese setting is unclear. Although PTMC is usually asymptomatic, several patients associate the idea of “living with cancer” with some degree of anxiety and experience a progressive decline in the quality of life.

### 3.3 Costs

Direct medical cost information was collected from a tertiary Chinese general hospital (**Supplementary Table 1**). These costs are controlled by the Chinese Government and show limited variation between institutions positioned parallelly in the healthcare system. Based on the Chinese treatment plan, monitoring costs were calculated by referring to real-world patient treatment cost data and consulting with clinical experts. All figures are provided in Chinese yuan (¥) (**Supplementary Table 2**).

## 4 Analysis

In agreement with the China Guidelines for Pharmacoeconomic Evaluations, we conducted the analysis using data from China’s healthcare system. According to these guidelines, we added a discount of 3% on future costs and benefits (23). We estimated the lifetime costs of two strategies and their effects in terms of QALYs (31). We calculated the incremental cost-effectiveness ratio, defined as the cost difference divided by the change in QALYs. The willingness-to-pay threshold (¥242,928) was estimated to be three times the gross domestic product per capita in China in 2021 (¥80,976). An incremental cost-effectiveness ratio of less than ¥242,928/QALY indicated that AS is cost-effective in China (32).

## 5 Results

### 5.1 Base case

In the base case scenario of a 40-year follow-up for both ES and AS (for patients diagnosed at 40 years of age), ES was costlier at ¥53,461, but it also had a greater effectiveness of 5.2 QALYs. In contrast, AS was more expensive at ¥74,198 and had an effectiveness of 25.8 QALYs. The effectiveness of treating patients with PTMC with AS was 20.6 QALYs, whereas the incremental cost per capita was ¥20,737. The corresponding incremental cost-effectiveness ratio was ¥1,009/QALY, which implied that for each additional QALY obtained, the incremental cost-effectiveness ratio was ¥1,009/QALY, which was lower than the willingness-to-pay threshold set for this study and lower than the per-capita gross domestic product in 2021. Therefore, even though AS was consistently more expensive than ES, it was also more effective (**Supplementary Figure 1**; **Table 2**).

**Table 2:** Cost-effectiveness of PTMC at different ages.

Follow-up time period	Age at diagnosis	Strategy	Cost, ¥	ΔCost	Effectiveness, QALY	ΔQALY	CER	ICER, ¥ per QALY
60-year HT vs 60-year AS	20	ES	53,450	N/A	5.2	N/A	10,261	N/A
		AS	137,744	84,294	29.8	24.5	4,626	3,431
40-year HT vs 40-year AS	40	ES	53,461	N/A	5.2	N/A	10,281	N/A
		AS	74,198	20,737	25.8	20.6	2,879	1,009
20-year HT vs 20-year AS	60	ES	53,449	N/A	5.2	N/A	10,279	N/A
		AS	38127	-15321	16.9	11.6	2256	-1316

HT, hemithyroidectomy; AS, active surveillance; ES, early surgery; QALY, quality-adjusted life-year; CER, cost-effectiveness ratio; ICER, incremental cost-effectiveness ratio; N/A, not applicable.

In the base case scenario of 20 years of follow-up for patients diagnosed at 60 years or more, even though AS was consistently more expensive at ¥137,744, it also had a higher effectiveness at 29.8 QALYs. The incremental cost-effectiveness ratio for AS was ¥3,431/QALY, which was lower than the willingness-to-pay limit. Owing to the difference in the initial monitoring age of patients, the net costs would be positive at 20 years and 40 years when AS was adopted. Meanwhile, from 60 years onwards, the annual net costs would be negative. At 60 years, AS was less costly than ES, at ¥38,127, and was more effective, with 16.9 QALYs achieved in older patients. ES was more expensive at ¥53,449 and was associated with 5.2 QALYs. The resulting incremental cost-effectiveness ratio for AS was −¥1316/QALY, which made AS cost-effective. We concluded that AS remained cost-effective regardless of the age at which patients were initially monitored (**Supplementary Figure 1**; **Table 2**).

### 5.2 Sensitivity analysis

**Supplemental Table 3** summarizes the results of the univariate sensitivity analysis for inputs that have limited effect on the incremental cost-effectiveness ratio in the reference case when the incremental cost-effectiveness ratio is lower than the threshold. These variables had the widest range in the incremental cost-effectiveness ratio when they varied from their greatest to their least range values. Two-way sensitivity analysis showed that varying the parameters did not substantially alter the cost-effectiveness of AS strategies (**Figure 3**).

**Supplemental Figure 2** depicts the results of the probabilistic sensitivity analyses. One hundred percent of the scatters were present in the first quadrant and were less than three times the gross domestic product per capita. This indicated that ES did not show cost-effectiveness in any of the 1,000 iterations. **Supplemental Figure 3** depicts the results of the cost-effectiveness acceptability curves. If the willingness-to-pay threshold increased to ¥24,292/QALY, AS would be cost-effective at a probability of 50%. If the willingness-to-pay threshold increased to ¥48,565.8/QALY, the probability of AS being cost-effective would increase to 100%.

## 6 Discussion

To our knowledge, this is the first study to examine the cost-effectiveness of AS in the management of incidental PTMC in China. In our reference case, AS was more cost-effective than ES throughout the lifetime of a patient with PTMC. However, findings from a prospective cohort study in Australia indicated that surgery may have a long-term economic advantage for younger Australian patients with PTMC (10); the study did not use a decision tree, Markov model, or utility score. Therefore, the validity of the model was low. This may explain the inconsistency with our findings. Additionally, one of the most frequently asked questions about AS is how long follow-up should continue and whether most of the processes are cost-effective over lifetime surveillance (33). Previous studies have estimated the cost-effectiveness based on 10-year or 20-year follow-ups, but patients with PTMC with a good prognosis usually survive for more than 20 or 30 years (17, 25). Therefore, even though our findings show that longer periods of follow-up may lead to higher costs for young patients, the incremental cost-effectiveness ratio (¥3,431/QALY) remains lower than the willingness-to-pay threshold over a 60-year follow-up period. Thus, we propose monitoring for patients with PTMC until the primary tumor size increases to 3 mm or more.

Particularly, when patients are selected for AS, their age should be considered. The findings of a multicenter cohort study in Korea indicated that the risk of an increase in the tumor volume in patients aged less than 45 years was twice greater than that of older patients who underwent AS for low-risk PTMC (34). Moreover, Lang et al. (17) showed in a subgroup analysis that patients aged less than 40 years prefer ES over AS. This suggests that an AS strategy according to age would be necessary in terms of not only cost but also effectiveness. Our research findings showed that AS always remained cost-effective at different initial monitoring ages. AS was found to be less cost-effective in younger patients and more cost-effective in patients with PTMC aged over 60 years. This result is concordant with the findings of a study conducted by Youssef et al. (35). The reason may be explained by the findings reported by Ito et al., showing that a greater age is associated with a lower risk of disease progression, tumor enlargement, and novel lymph node metastasis. In such cases, AS may limit the need for ES and thereby decrease surgical complications (36). Another reason may be that PTMC occurs at an earlier age than other cancers (37), and younger patients tend to exhibit a greater risk of disease progression than elderly patients (27).

When the patient was younger than 60 years, the AS strategy was more expensive, and the total cost increased more rapidly than that of ES. Therefore, when only cost was considered, AS was preferable for patients at an advanced age or with a reduced (20 years) life expectancy, whereas ES was preferable for younger and healthier patients (with a longer life expectancy). This could be attributed to the continuance of half-yearly examinations and the cumulative possibility of more costly “late” surgery and the associated complications over time. In a similar study, Kim et al. (38) reported that the initial cost of AS is estimated to be 5.6 times lower than that of lobectomy, whereas the 10-year cumulative costs of AS ($2,545) and lobectomy, regardless of LT4 ($3,045), are similar at a discount rate of 3%. However, in the long-term follow-up period, immediate surgery is estimated to be more economical than AS. The costs of the two management approaches are similar in Hong Kong (17), wherein adopting the non-surgical approach not only ensured cost-effectiveness in the initial 16 years but also ensured that the method remained cost-effective thereafter. This finding was substantially different from that in the United States and Japan, implying that the outcome could be affected by each country’s national health insurance coverage and the thyroid ultrasound interval during follow-up.

However, in terms of effectiveness, as measured by QALY gained, AS was more effective than ES, regardless of the length of observation. This was because the former resulted in fewer permanent procedure-related complications. In fact, in the sensitivity analysis, incremental QALYs were always positive, implying that AS was always more effective than ES, regardless of surgical complications, the rate of recurrence, or the discount rate. Furthermore, the range of the incremental cost-effectiveness ratio was far lesser than the willingness-to-pay threshold. Therefore, consistent with the findings from our initial hypothesis, AS is always a cost-saving or cost-effective strategy in PTMC management. In addition, patient preference and willingness to participate in AS may be difficult to predict, and some patients may abandon AS owing to anxiety associated with the burden of living with cancer. Patient compliance may be reduced during the years of follow-up. Additionally, the convenience of the clinical consultation environment is often not convincing for Chinese patients. Furthermore, the medical billing value for AS management cannot match that of a physician (usually ¥7 per outpatient visit), which makes follow-up during AS challenging. To promote the implementation of AS in China, it may be necessary for an authoritative thyroid surgeon to provide reasonable communication and follow-up and to tailor AS strategies for patients with low-risk PTMC. Additionally, reforming the medical billing system for diagnosis and treatment is essential to fully encourage doctors.

The considerably low cost of outpatient services in China made the differences between the costs of ES and AS seem obvious. The ES cost-effectiveness ratio is twofold greater than the AS cost-effectiveness ratio at 20 years of age. Among individuals and families, 21% of cancer patients met the WHO standard of poverty owing to illness (healthcare costs >30% of the household income) in China. For the government, treating a disease with an excellent prognosis may require the use of financial resources in large amounts owing to overdiagnosis and overtreatment. If approximately 50,000 patients are over-diagnosed with PTMC in China in 2022, with each patient aged 40 years or more and opting for AS, the government can save ¥10 × 10^8^ (8). Notably, the actual circumstances may be more severe than this. Therefore, real-world investigations on the cost-effectiveness of PTMC based on data from the Chinese population and the incorporation of AS into the management plan for PTMC in China should be implemented as soon as possible.

The differences between the findings of this study and previous studies may be partly attributed to the differences in the treatment styles adopted in different countries. First, owing to insufficient radiofrequency ablation, the challenges of radiofrequency ablation after surgery have increased. The treatment method used in our study did not involve radiofrequency ablation; instead, we used methods recommended in the standard clinical process for PTMC treatment in China. Additional prospective studies and high-level evidence-based medical data for long-term follow-up observations are needed to demonstrate the safety and efficacy of AS in clinical settings. In addition, because PTMC is mostly an early tumor, the unilateral resection of the glandular lobe and isthmus is sufficient to remove malignant tissue. Thus, postoperative radioiodine remnant ablation is unnecessary. Current consensus also does not recommend radiofrequency ablation and radioiodine remnant ablation in low-risk patients. Second, because only a small number of patients with PTMC develop lymph node metastasis, lymph node dissection in zone VI on the side of the lesion should be performed during hemithyroidectomy + central neck dissection and total thyroidectomy + central neck dissection, with careful dissection and effective preservation of the parathyroid gland and recurrent laryngeal nerve (9). Owing to the rarity of lateral lymph node metastasis with PTMC, lateral lymph node dissection was not performed in the hemithyroidectomy + central neck dissection and total thyroidectomy + central neck dissection procedures. Third, in the United States, the follow-up interval for thyroid ultrasound is 1 year, whereas, in Hong Kong, it is 6 months. In this study, the frequency of follow-up was decided according to the diagnosis and treatment norms in the consensus. The follow-up interval in the two groups of patients with ES and AS was 6 months, which may have affected the results of the cost-effectiveness analysis and the generalizability of findings. The best imaging method for regular follow-up was thyroid ultrasound, which is usually performed by a specialized radiologist using standard specialization (17, 25). The use of ultrasound for measuring thyroid tumor size and lymph node metastases may be challenging. However, ultrasound and pathology have been used successfully to measure tumors only a few millimeters apart in length with precision. The accuracy of an ultrasound diagnosis depends on the experience of the technician, and considerable differences may exist between the results obtained by ultrasound technicians with different levels of expertise (11).

Our study had several limitations. First, insurance policies and medical costs vary in different countries, and the costs in this study are likely to differ from those in other countries. In our study, we only considered direct medical costs but excluded direct non‐medical costs and indirect costs (unavailable). Hence, we could not determine cost-effectiveness from a societal perspective, which is the most appropriate and comprehensive perspective. Additionally, because the cost of adverse reactions was included in hospital costs in the base case scenario, we only considered the most important complications (such as hypothyroidism, hypoparathyroidism, and unilateral/bilateral recurrent laryngeal nerve injury) but not the total cost of adverse reactions. Undoubtedly, a part of the cost of adverse reactions may have been excluded. However, if these adverse effects are considered in the study, the difference in cost-effectiveness between AS and ES may be even greater, suggesting that AS is more cost-effective for PTMC. Furthermore, the cost-effectiveness of AS versus ES in the context of the Chinese healthcare system has not been considered in other studies. Therefore, the transformation probability and health utility refer to those in similar foreign studies. This also suggests the need for further prospective studies, including assessments of the QALY scores of patients with PTMC, to ensure that the right measures are taken while preparing patient-tailored treatment plans. Additionally, because these parameters change as patients age, fixed inputs, such as the probability of recurrence after HT and total thyroidectomy, were limitations of this study. However, the results of the sensitivity analysis showed that the value was robust over a relatively wide range of inputs. Finally, because this study was only based on a mathematical model, we should have considered various local factors in China (e.g., surgical modality, regular monitoring programs, and the national healthcare system) when selecting the best management strategy for Chinese patients with PTMC. The results are applicable only to the Chinese healthcare system and should be interpreted cautiously in other countries.

## 7 Conclusions

AS was a more cost-effective strategy for patients with PTMC than ES and remained cost-effective at different initial monitoring ages. The findings of this study provide essential evidence for China’s PTMC management policy. In China, the overtreatment of PTMC leads to unfavorable changes in the balance between patient benefits and the economy, which is an early warning sign for this emerging economy and other countries at similar stages of development.

## Data availability statement

The original contributions presented in the study are included in the article/**Supplementary Material**. Further inquiries can be directed to the corresponding author.

## Author contributions

ML, MZ, QQ, YA, and YL collected data. ML, MZ, QQ, and WY analyzed the data. ML, MZ and QQ wrote the manuscript. WY approved the manuscript. All authors contributed to the article and approved the submitted version.
