# Supplementary material for: Cost-effectiveness of active surveillance versus early surgery for thyroid micropapillary carcinoma based on diagnostic and treatment norms in China
Source: Front Endocrinol (Lausanne). 2023 Aug 16;14:1166433. doi: 10.3389/fendo.2023.1166433 (PMC10471146; doi:10.3389/fendo.2023.1166433)

SUPPLEMENT TABLE 1. Costs

Inspection Item	Cost(¥)
Physician office visits	7
Fine-needle aspiration cytology	762
Ultrasonography	60
Chest CT	510
CT neck enhancement scan	625
Electrocardiogram	20
Laryngoscopy tests	210
Bone density tests	360
Blood tests	219
Thyroid function	273
24-hour urine calcium, urine phosphorus	34
Parathyroid hormone tests	52
Drugs of Hypothyroidism(one year)	175
Drugs of Hypoparathyroidism(one year)	2850
Drugs of recurrent laryngeal nerve injury(one year)	2400
Unilateral recurrent laryngeal nerve repair surgery	1000
Bilateral recurrent laryngeal nerve repair surgery	2000

SUPPLEMENT TABLE 2. Model inputs for costs

Input	Reference case value（¥）	Distribution	Analysis range	Mean	Standard deviation
HT+ CND	15204	Normal	13684-16724	15204	776
TT+ CND	22600	Normal	20340-24860	22600	1153
Recurrence post-HT	22600	Normal	20340-24860	22600	1153
Redo LND	4188	Normal	3769-4607	4188	214
AS stable disease	1158	Normal	1042-1274	1158	59
AS	1158	Normal	1042-1274	1158	59
Temporary complication post-HT	1620	Normal	1458-1782	1620	83
Permanent complication post-HT	5022	Normal	4520-5524	5022	256
No complication post-HT	2353	Normal	2118-2588	2353	120
Permanent hypoparathyroidism post-TT LND	5511	Normal	4960-6062	5511	281
Permanent unilateral RLN injury post-TT LND	6173	Normal	5556-6790	6173	315
Permanent bilateral RLN injury post-TT LND	7273	Normal	6546-8000	7273	371
No complication post-TT	2408	Normal	2.167-2.649	2408	123

SUPPLEMENT TABLE 3. Results of univariate sensitivity analysis

Parameters	Parameter range	Range for incremental QALYs		Range for ICER	
**Probability**					
Permanent complication after HT	0.014-0.017	24.570	24.570	3430.475	3430.912
Temporary complication after HT	0.014-0.017	24.570	24.570	3430.767	3430.767
Recurrence after lobectomy	0.0036-0.0044	29.779	29.779	3428.095	3433.429
Recurrence after total thyroidectomy	0.007-0.009	24.570	24.570	3430.608	3430.925
Stable disease after HT	0.616-0.752	23.276	25.435	3051.676	3662.649
Permanent unilateral RLN injury after TT	0.014-0.017	24.570	24.570	3430.469	3431.361
Permanent hypoparathyroidism after TT	0.080-0.098	24.570	24.570	3428.797	3432.736
Permanent bilateral RLN injury after TT	0.0027-0.0033	24.570	24.570	3430.627	3430.907
Death after HT	0.0018-0.0022	24.558	24.581	3430.690	3430.834
Death after TT	0.0018-0.0022	24.561	24.579	3430.716	3430.818
Complication after redo LND	0.288-0.352	24.570	24.570	3430.720	3430.813
**Costs**					
AS stable disease	1042.4-1274	24.57	24.57	3333.141	3528.561
Permanent complication after HT	4519.8-5524	24.567	24.567	3430.486	3431.048
Temporary complication after HT	1458-1782	24.567	24.567	3430.668	3430.866
No complication after HT	2117.7-2588	24.567	24.567	3422.228	3439.317
Permanent unilateral RLN injury after TT	5,555.7-6,790	24.567	24.567	3430.149	3431.384
Permanent bilateral RLN injury after TT	6,545.7-8,000	24.567	24.567	3430.621	3430.912
Permanent hypoparathyroidism after TT	4,959.9-6,602	24.567	24.567	3427.495	3437.243
Redo LND	3,769.2-4,607	24.567	24.567	3430.697	3430.836
Complication after TT	2167.2-2649	24.567	24.567	3416.457	3445.089
Active surveillance	1042.2-1,274	24.567	24.567	3296.276	3565.258
Early surgery	13683.6-16,724	24.567	24.567	3243.79	3617.793
Nodal metastasis	20,340-24,860	24.567	24.567	3275.113	3586.42
Primary tumor growth	13683.6-16,724	24.567	24.567	3297.347	3564.152
Recurrence	20,340-24,860	24.567	24.567	3430.428	3431.106
**Health utilities**					
AS stable disease	0.891-1	22.520	24.777	3402.085	3743.182
No complication after HT	0.891-1	24.561	24.656	3418.469	3432.014
Permanent complication after HT	0.567-0.693	24.57	24.57	3430.646	3430.888
Temporary complication after HT	0.5643-0.6897	24.57	24.57	3430.635	3430.898
Permanent bilateral RLN injury after TT	0.189-0.231	24.57	24.57	3430.752	3430.781
Utilities of permanent hypoparathyroidism after TT	0.7002-0.8558	24.56	24.58	3429.183	3432.352
Permanent unilateral RLN injury after TT	0.5651-0.6907	24.57	24.57	3430.551	3430.982
Redo LND	0.504-0.616	24.57	24.57	3430.667	3430.867
LND complication	0.369-0.451	24.57	24.57	3430.790	3430.743
No complication after TT	0.747-0.913	24.449	24.691	3413.928	3447.773
Active surveillance	0.099-0.121	24.298	24.842	3393.199	3469.175
Disease progression	0.486-0.594	24.57	24.57	3430.679	3430.855
Early surgery	0.666-0.814	24.347	24.793	3399.938	3462.159
Nodal metastasis	0.225-0.275	24.486	24.653	3419.136	3442.477
Primary tumor growth	0.486-0.594	24.341	24.799	3399.022	3463.110
Recurrence	0.486-0.594	24.57	24.57	3430.739	3430.794
Discount rate	0.01-0.05	15.555	42.485	3253.346	3528.471

SUPPLEMENT FIGURE1.Cost-effectiveness e analysis in 20-y, 40-y, 60-y ES vs 20-y, 40-y, 60-y

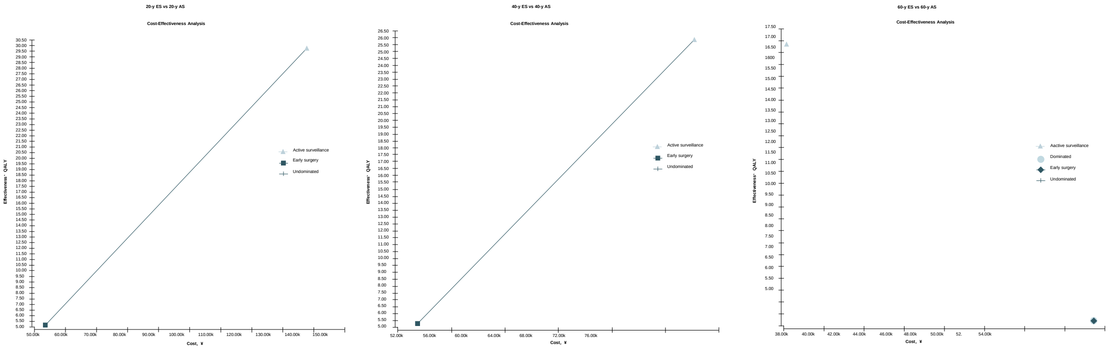

SUPPLEMENT FIGURE2. Probabilistic sensitivity analysis

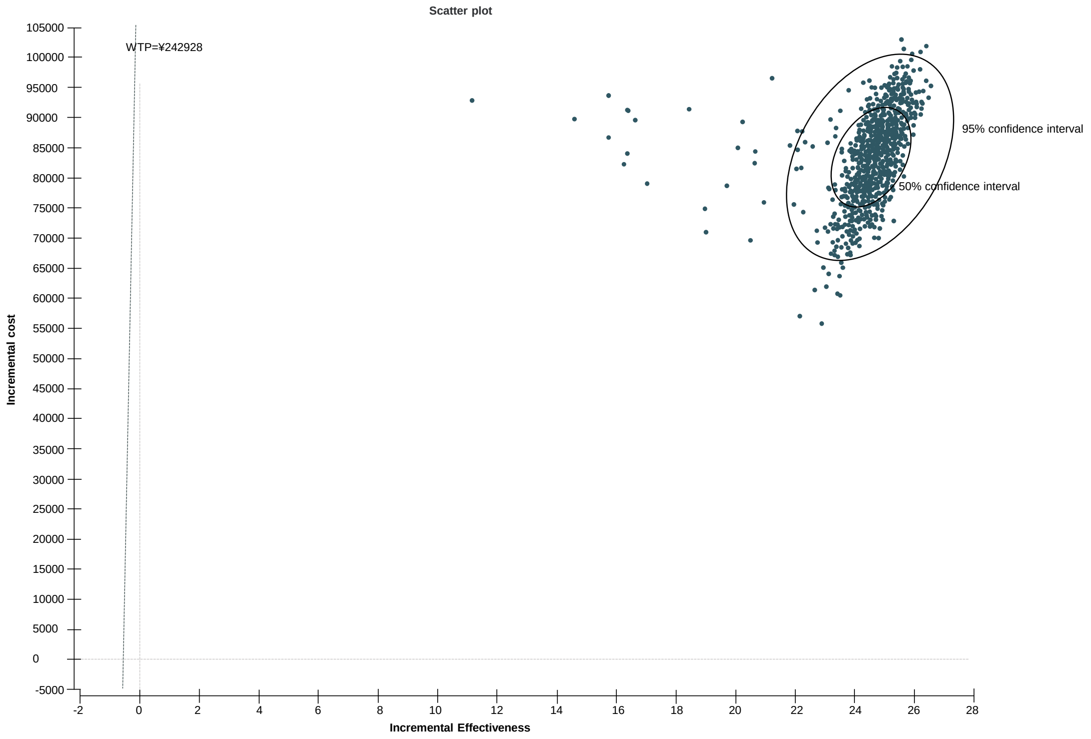

SUPPLEMENT FIGURE 3. Cost–effectiveness acceptability curves

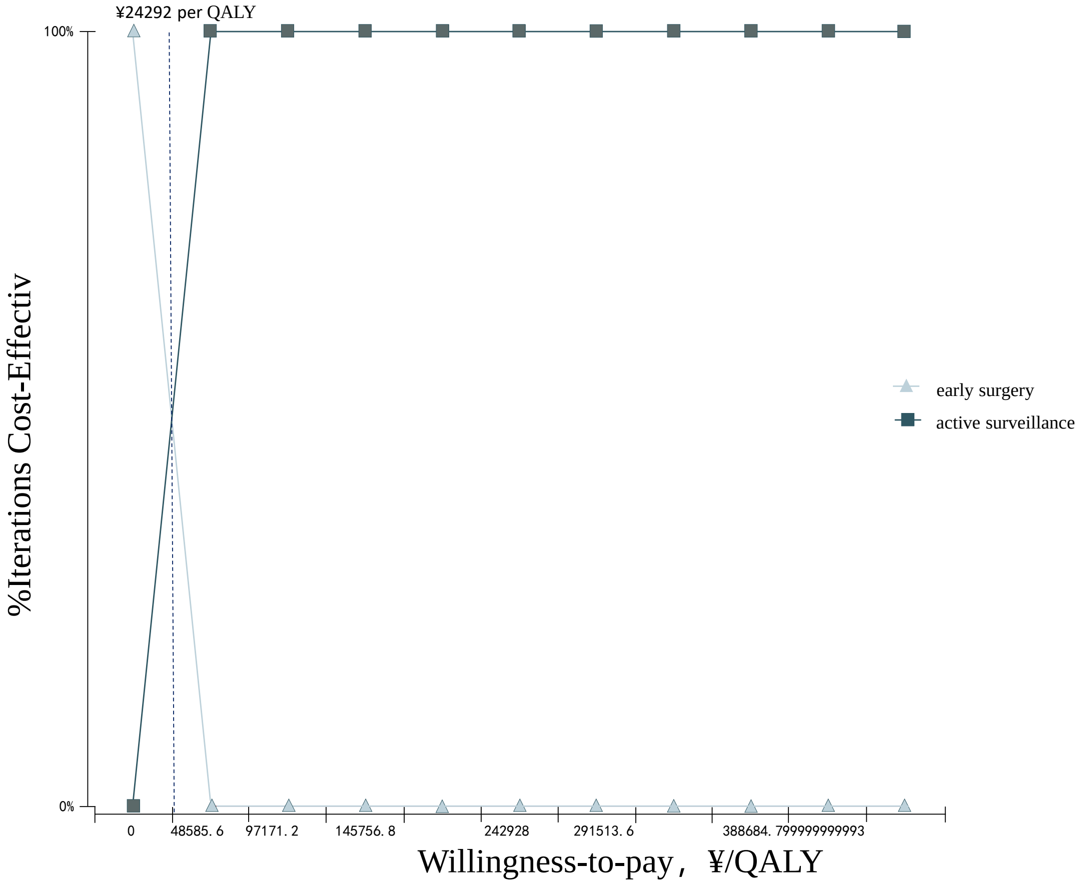

Supplement: Supplementary file 1 [file DataSheet_1.pdf]
